# Characteristics of Aqueous Chemical Species Generation in Plasma‐Facing Liquid Systems Using Helium Jet Plasma

**DOI:** 10.1002/open.202300213

**Published:** 2024-05-27

**Authors:** Joo Young Park, Jin Hee Bae, Seunghun Lee

**Affiliations:** ^1^ Nano-Bio Convergence Division Korea Institute of Materials Science 797 Changwondae-ro Changwon 51508 Republic of Korea; ^2^ Present address: Department of Nuclear and Quantum Engineering Korea Advanced Institute of Science and Technology 291 Daehak-ro, Yuseong-gu Daejeon 34141 Republic of Korea

**Keywords:** helium plasma jet, long-lived species, plasma chemistry, radicals, RPMI 1640

## Abstract

Plasma‐facing liquids (PFLs) facilitate the storage of reactive O and N species (RONS), including H_2_O_2_ and NO_2_^−^, which remain in the PFL after plasma treatment, and they can continuously influence the target immersed in the liquid. However, their behaviors and levels of generation and extinction depend strongly on the plasma characteristics and liquid condition. Therefore, understanding the effects of the liquid type on the plasma discharge characteristics and the RONS generated via plasma discharge is necessary. We compared the RONS generation and storage trends of deionized H_2_O and a high‐conductivity PFL, RPMI 1640, which is a well‐known cell culture medium commonly used to culture mammalian cells. RPMI 1640 acted as an electrode and enhanced the plasma discharge power by supplying abundant radicals and RONS. The production of gaseous hydroxyl radicals and NO markedly increased, which facilitated H_2_O_2_ and NO_2_^−^ production in the PFL for the first 200 s, and then the increase in the RONS concentration stagnated. With respect to storage, as the components within RMPI 1640 exhibited high reaction constants for their reactions with H_2_O_2_, H_2_O_2_ elimination was completed in <30 min. Unlike H_2_O_2_, the concentration of NO_2_^−^ in the PFL was unchanged.

## Graphical abstract

Ion layers in cell culture media induce larger amounts of NO and OH radicals in plasma‐facing liquids compared to those in deionized H_2_O, whereas scavengers reduce H_2_O_2_ during the post‐discharge period.

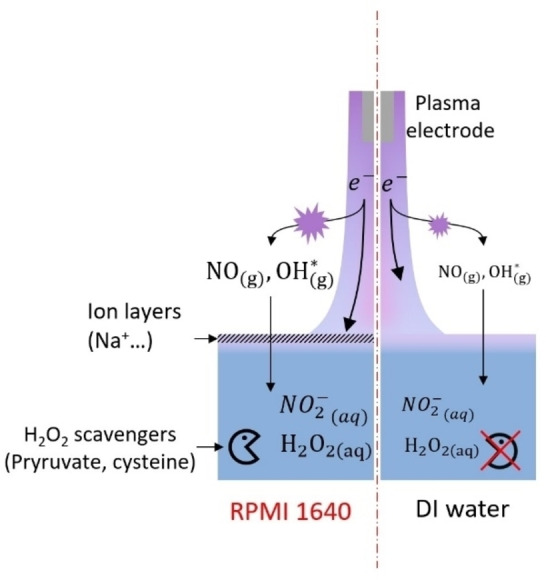

## 1 Introduction

Atmospheric‐pressure plasma technology is widely applied in the fields of semiconductor fabrication, gas reforming, and biomedical technology and agro‐food development, which exploit the capacity of plasma to generate various active species using only N_2_ and O_2_ in open air.[^1^, ^2^, ^3^, ^4^, ^5^, ^6^, ^7^] Molecular N_2_ and O_2_ are stable; however, when they are separated into atoms or ions, the highly reactive radicals significantly reduce the mean free paths of electrons and may cause difficulties in glow discharge.[^8^, ^9^] When an inert gas is added, the mean free path within the plasma is extended. Hence, the inert gas exhibits a lower probability of consuming energy via vibrational and rotational excitation, dissociation, and excitation to a metastable state, even in the plasma state, compared to those of other gases. As it consumes less power, this is a good method of stably operating plasma with less power compared to that when using a non‐inert gas. The advantage of low power consumption enables the maintenance of the plasma at a low temperature, allowing its use in biological fields where high temperatures must be suppressed.[^10^, ^11^, ^12^]

The atmospheric‐pressure plasma jet (APPJ) one of the representative atmospheric‐pressure plasma device that is mainly applied in the biomedical field,[^13^, ^14^, ^15^, ^16^] and its discharge area can be freely controlled by tuning the flow rate of the noble gas. Although applying the APPJ over a large area is challenging because of its nozzle size and the high costs of noble gases, it displays noticeable effects in various small‐scale fields, such as cancer cell death, cell mobility inhibition, bacterial sterilization, cell function improvement, and the treatment of the skin and psoriasis.[^17^, ^18^, ^19^, ^20^, ^21^] In these applications, numerous research groups apply the plasma jet through cell‐containing media or the epidermis surrounding the cells, and thus, the effect of the plasma is transmitted to the cell through cell‐containing media.

Plasma‐facing liquids (PFLs), one type of plasma‐activated liquid, have attracted considerable research attention in terms of the elucidation of its physicochemical properties and the expansion of its application scope. Plasma‐activated liquid refers to all liquid affected by plasma discharge, while PFL has the advantage of being more efficient in that it can supply short‐lived chemical species directly to the liquid surface because the plasma discharge plume faces the liquid surface.[^22^, ^23^, ^24^, ^25^, ^26^] In particular, when using inert gas such as He as discharge gas, it has the great advantage of being able to keep the temperature of the plasma jet low, so it is particularly strong in the bio‐field where maintaining an appropriate temperature is required. However, in the case of PFL, since the plasma plume contact with the liquid surface, the discharge characteristics also change depending on the state of the liquid, and it cannot be overlooked that the reactive oxygen and nitrogen species (RONS) also change eventually.[^27^, ^28^, ^29^]

RONS in the PFL are generally considered highly significant intermediates in the field of biomedical research. Reactive O species (ROS), such as the hydroxyl radical (OH*), typically oxidize the components in a liquid, stimulate the cells, and deactivate viruses and bacteria, whereas reactive N species (RNS), such as NO, act as signaling chemicals between groups of cells.[^30^, ^31^, ^32^, ^33^] Plasma directly supplies RONS, such as OH or NO, denoted short‐lived species, which are only effective for short periods. Although these species exhibit strong short‐term effects, they are rapidly converted to long‐lived species, such as NO_2_^−^ and H_2_O_2_. Additionally, they are well‐known effective species in PFL systems.^[34]^ NO_2_^−^ and H_2_O_2_ remain in the PFL after the completion of plasma treatment, and they can continuously influence the target immersed in the liquid. However, their behaviors and levels of generation and extinction depend strongly on the plasma characteristics and liquid condition. Therefore, understanding the role of the type of liquid in terms of the plasma discharge characteristics and behaviors of the RONS generated via plasma discharge is necessary. O_3_ is also a well‐known effective RONS; however, the treatment duration is insufficient for it to dissolve in solution in a PFL system.

In order to utilized APPJ as PFL system efficiently, it is important to understand the characteristics of the APPJ itself, but is also very significant to understand the characteristics of the plasma and target liquid that vary depending on the target liquid type. Especially, to avoid a very easy misunderstanding that the same APPJ is expected to have the same effect regardless liquid condition, we prepared two type of solutions and evaluated their characteristics.

In this study, we interpreted the discharge shapes based on the changes in the concentrations of H_2_O_2_ and NO_2_^−^ in a PFL, deionized (DI) H_2_O, and RPMI 1640, which is a cell culture medium commonly used to culture mammalian cells.[^35^, ^36^, ^37^] As the RPMI 1640 solution contains various nutrients, such as vitamins, glucose, and amino acids, numerous reactions with plasma‐generated chemical species were expected. An He‐based APPJ device was operated at a low power (<4 W) with DI H_2_O and RPMI 1640. Optical emission spectroscopy (OES) was used to investigate the dissipated power with respect to the type of liquid used. The saturation of RONS was investigated using ordinary differential equation (ODE)‐based numerical chemical calculations that considered all chemical reactions of candidate RONS scavengers. The conductivity of the liquid controls the discharge properties in a PFL system, and we explained the increases in the concentrations of NO_2_^−^ and H_2_O_2_ to saturation based on the enhancement in the dissipated power. Saturation was attributed to the presence of H_2_O_2_ scavengers within the RPMI 1640 solution.

## Experimental Section

### PFL System

**Figure 1:**
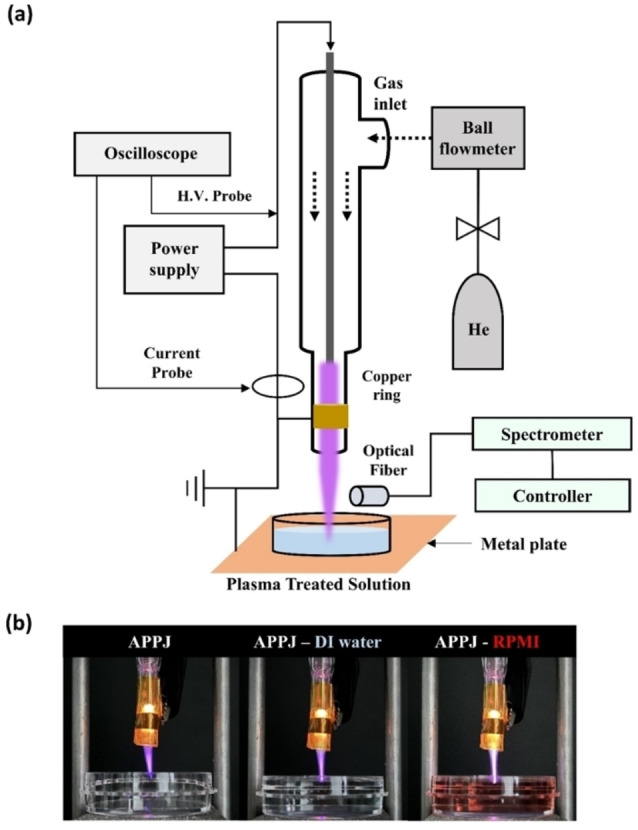
(a) Illustration of the PFL preparation system with the APPJ. (b) Images of the APPJ under three conditions (ambient air, DI H_2_O, and RPMI 1640).

A schematic of the experiment is shown in Figure 1, including plasma discharge images depending on the liquid type. A high‐voltage electrode (diameter: 2 mm) was located within the Pyrex tube (inner diameter: 4 mm, outer diameter: 6 mm), and a Cu‐tape ground electrode (width: 5 mm) is wrapped around the Pyrex tube. He gas (99.99 % purity) is supplied at a flow rate of 3 L/min through a gas inlet using a ball flow meter. The lab‐developed power source driven by a sine wave displays a fixed driving voltage and frequency of 4.8 kV and 37 kHz, respectively. The electrical characteristics are measured using high‐voltage differential (SI‐9010, Sapphire Instruments, Ahmedabad, India) and current probes (Model 4100 C, Pearson Electronics, Palo Alto, CA, USA) and recorded using a digital oscilloscope (DPO3034, Tektronix, Beaverton, OR, USA). OES was performed over the ultraviolet‐visible range by using a spectrometer (Maya2000Pro, Ocean Optics, Dunedin, FL, USA), and the collecting lens (UV‐74, Ocean Optics) for spectral focus is located 5 mm below the nozzle. Images of the plasma discharge are captured using a DSLR camera (EOS 5D Mark IV, Canon, Tokyo, Japan).

### Experimental Arrangement

The PFL preparation system using the APPJ device for model experiments was constructed as follows. The liquid sample (3 mL) in a Petri dish with a diameter of 30 mm was located 8 mm below the exit of the APPJ source. DI H_2_O was prepared using deionizing equipment (Pure Power 1, Human, Seoul, Republic of Korea), and RPMI 1640 solutions (LM 011‐51, Welgene, Gyeongsan, Republic of Korea) was prepared in empty Petri dishes to evaluated to understand the unique APPJ characteristics. To investigate the properties of the liquids used, the pH values and conductivities of the plasma‐activated liquids were measured using pH (Seven2Go pH meter S2, Mettler Toledo, Columbus, OH, USA) and conductivity meters (CM‐3, CAS, Yangju, Republic of Korea), respectively. The APPJ temperature was also measured for safe biomedical application, and it was maintained at <40 °C. The liquid was maintained at <35 °C for a discharge time of 20 min.

### Quantification of Plasma Properties and RONS within the Liquid (H_2_O_2_, NO_2_^−^)

#### Excitation Temperature (T_exc_)

The T_exc_ of the He jet plasma was obtained via the Boltzmann plot method. This analytical method calculates the T_exc_ based on the ratio of the peaks corresponding to He in the OE spectrum of the plasma.^[38]^

#### H_2_O_2_ in the PFL

The concentration of H_2_O_2_ was measured using NH_4_VO_3_, which is free from the disturbances caused by NO_2_^−^ and NO_3_^−^. The peroxovanadium cation (VO_3_^3+^), which absorbs light at 450 nm, is produced by the reaction of H_2_O_2_ with NH_4_VO_3_ under acidic conditions, as follows:^[39]^

(1) $${VO}_{3}^{-}+4H^{+}+H_{2}O_{2}\to {VO}_{2}^{\;3+}+{3H}_{2}O\;$$

The measurement methods, including the preparation of the standard solution, followed the protocol described by Park et al.^[34]^

#### NO_2_^−^ in the PFL

An NO_2_^−^ concentration of <0.1 mM in a Petri dish is challenging to measure quantitatively via in‐situ optical absorption spectroscopy. The Griess assay can be used to analyze NO_2_^−^ and enables the effective concentration measurement of N oxides, even at low concentrations.^[40]^ Sulfanilic acid was quantitatively converted to a diazonium salt via a reaction with NO_2_^−^ in an acidic solution. The diazonium salt was then coupled to *N*‐(1‐naphthyl) ethylenediamine, forming an azo dye, which absorbed 548 nm light. The absorption spectrum was recorded using a microplate reader (SPECTROstar Nano, BMG LABTECH, Ortenberg, Germany).

## 2 Results and Discussion

### 2.1 Background of APPJ and RONS Production in PFL

The generation of NO_2_^−^ and H_2_O_2_ in the He‐PFL via the ambient‐air discharge plasma at a low electron temperature (T_e_) has been studied extensively.[^41^, ^42^, ^43^, ^44^, ^45^] The major pathways of these RONS are described in this section.

Most chemical reactions are based on the electron produced from He gas or excited He. The following reactions mainly occur in a plasma plume with a relatively high He concentration:

(2) $$He+e\to {He}^{+}+2e$$

(3)

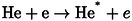




As the plasma plume penetrates the ambient air, excited He begins to react with the nitrogen and oxygen surrounding the plasma plume as follow:
(4)

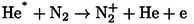



(5)

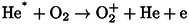




Not only excited He, electron produced He also affect nitrogen, oxygen and water vapor ionization producing RONS.
(6)

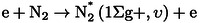

(7) $$e+O_{2}\to 2O^{\circ }+e$$

(8)

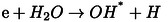




The products of reaction 6–8 are the major sources of NO_2_^−^ and H_2_O_2_ production.

#### 2.1.1 NO_2_^−^

When the plasma discharges, highly excited N_2_ reacts with atomic O, producing NO and atomic N in the gaseous phase (O+N_2_*(^1^Σ_g_^+^,υ)→NO+N), and atomic N becomes a source of NO via the reaction with O_2_ gas (N+O_2_→NO+O). Atomic O is generated via O_2_ dissociation caused by electron impact (O_2_+e→2O+e^−^), and it is a good source for use in producing strong oxidants, such as O_3_ or OH (e. g., O+O_2_→O_3_). These oxidants react with NO, producing N oxides (N_x_O_y(g)_). Gaseous nitrous acids (HONO) are formed by N_x_O_y(g)_, which reacts with H_2_O vapor (e. g., N_2_O_3(g)_+H_2_O_(g)_→2HONO_(g)_), and they are dissolved into the liquid (e. g., HONO_(g)_→HONO_(aq)_).

In conclusion, as NO generation in the gaseous plasma discharge layer is the first process in the NO_2_^−^ generation pathway, the NO_2_^−^ concentration in the PFL is highly proportional to the NO_(g)_ concentration.

#### 2.1.2 H_2_O_2_

The generation pathway of H_2_O_2_ in the PFL is considerably simpler than that of NO_2_^−^. As mentioned above, The major reaction is the combination of hydroxyl radicals (OH*), which originate from the electron‐impact dissociation of H_2_O (H_2_O+e^−^→OH*+H). The resultant OH^*^ species can directly dissolve in the liquid or combine in the gaseous phase. OH^*^ species dissolved in the liquid also combine to form H_2_O_2_.

### 2.2 Enhancement of Discharge Energy due to Electrical Conductivity of PFL

**Figure 2:**
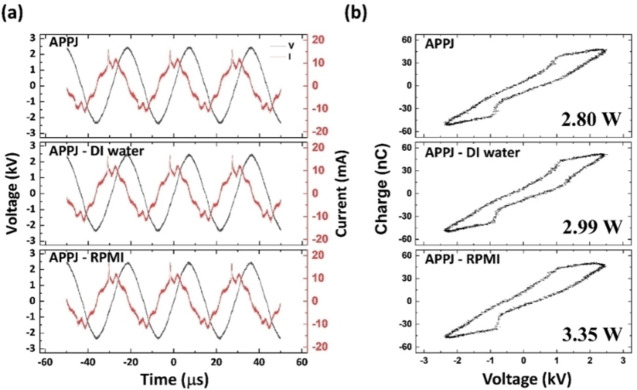
(a) V–I graphs and (b) Lissajous curves of the APPJ operating system under three conditions (ambient air, DI H_2_O, and RPMI 1640).

As shown in Figure 1b, the shape of the plasma discharge clearly changes, even to the naked eye, depending on the type of liquid. In the figure, “APPJ” denotes the APPJ without a liquid in the Petri dish and “APPJ–DI H_2_O” and “APPJ–RPMI” respectively denote the PFL systems with DI H_2_O and RPMI 1640. Figures 2a and b show the electrical properties, including the V–I and Lissajous curves, respectively.

The V–I characteristic may be independent of the liquid type; however, the dissipated power depends on the liquid type. APPJ–RPMI displays a higher dissipated power than that of APPJ–DI H_2_O, which exhibits a higher dissipated power than that of APPJ.

**Figure 3:**
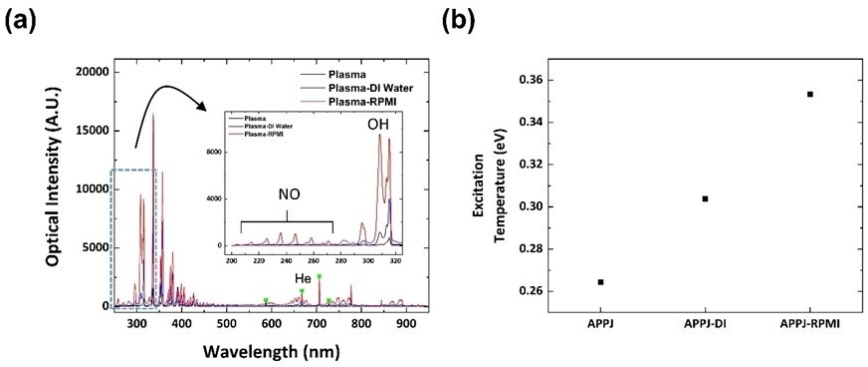
(a) Optical emission spectrum of the APPJ based on the liquid conditions and the (b) excitation temperatures of the three systems.

The liquid type changes not only the dissipated power but also the OE spectrum, as shown in Figure 3a. Clearly, the optical intensity increases with dissipated power. The two critical parameters are the NO band (220–260 nm) and OH peak (309 and 315 nm). The OH peak is intense, weak, and almost nonexistent in the spectrum of plasma/RMPI, plasma/DI H_2_O, and plasma only, respectively. A similar trend is observed for the NO band.

This trend may be attributed to the T_exc_. As T_exc_ describes the population of the excited energy levels based on the Boltzmann distribution, it indicates the number of excited atoms or molecules. Figure 3b shows the trend of T_exc_, as obtained via the ratio of the

He atomic lines, in terms of the liquid type.^[46]^ T_exc_ generally increases by approximately 0.04–0.05 eV in the order of APPJ, APPJ–DI, and APPJ–RPMI. A high T_exc_ in the discharge layer suggests more‐excited N_2_, which is a major source of NO production, and thus, the NO band is stronger at a high T_exc_. Moreover, in weakly ionized plasma, T_exc_ is proportionally related to T_e_, and thus, the electron‐impact H_2_O dissociation of APPJ–RPMI is enhanced.

**Figure 4:**
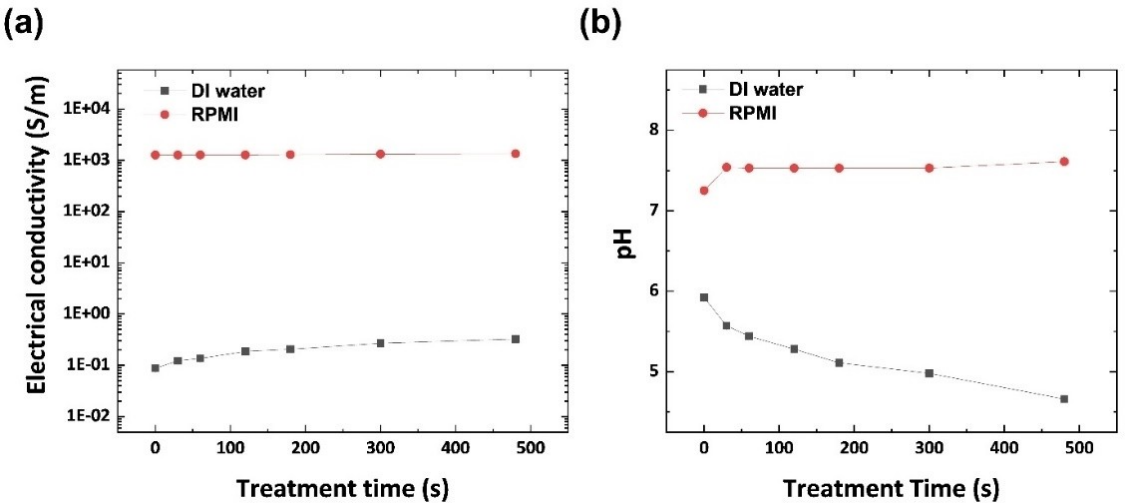
(a) Electrical conductivities and (b) pH values of two types of PFLs (black: DI H_2_O, red: RPMI 1640) as functions of treatment time.

The rationale for the higher T_exc_ and stronger OES peak intensities (NO band and OH peak) of APPJ–RPMI is explained by Figure 4. Figures 4a and b show the temporal trends of electrical conductivity and pH, respectively. The electrical conductivity of RPMI 1640 is 10^4^‐fold higher than that of DI H_2_O. Although the electrical conductivity of DI H_2_O increases because of ion generation within the PFL, it is considerably lower than that of RPMI 1640. Although ion generation is stronger in RPMI 1640, it apparently exhibits a negligible effect on the already‐high electrical conductivity. A comparison of the electrical conductivity of the PFL with those of other well‐known materials indicates that the RPMI 1640 PFL (σ≈1×10^3^ S/m) displays an electrical conductivity similar to that of amorphous C (σ≈1.5×10^3^ S/m)^[47]^ and higher than that of Ge (σ≈2.17×10^−2^ S/m).[^48^, ^49^] Amorphous C can be used as an electrode material, and Ge is commonly used in semiconductors, and thus, the RPMI‐1640‐based PFL can also be used as an electrode material, unlike the DI‐H_2_O‐based PFL. Thus, the electrical channel between the plasma plume and RPMI 1640 surface is strongly connected, i. e., plasma bullets and ions in the plasma plume accelerate and display high energies, and thus, they can be more actively involved in RONS generation. These phenomena also induce strong ion flows, and thus, the population of reactive species in the plasma plume involved in RONS generation, particularly OH and NO radicals, is increased.

Commonly, the generation of HNO_3_, with a pKa of less than −1 in the aqueous phase, during plasma discharge in the PFL lowers the pH, as shown in Figure 4b.[^50^, ^51^] Due to the pH buffering effects of the RPMI 1640 solution, the change in the pH is negligible. Although pH alone cannot be used to measure the total ion concentration (pH indicates only H ions), it should be related to the role of the electrode on the liquid surface, because it is related to the ionization rate, e. g., HONO, which is a conjugate of NO_2_^−^ (HONO↔NO_2_^−^+H^+^), displays a pKa of 3.3–3.8.[^52^, ^53^, ^54^] A higher pH indicates a higher ionization rate for the formation of NO_2_^−^ and H^+^. After 400 s of plasma treatment, the pH of the DI‐H_2_O‐based PFL decreases to ~4. This suggests that the ionization rate of HONO remains at almost 60–80 %, whereas HONO is completely ionized at pH 7.4 (RPMI 1640). These phenomena can also affect the electrical conductivity.

### 2.3 RONS (H_2_O_2_, NO_2_^−^) Production in PFL

**Figure 5:**
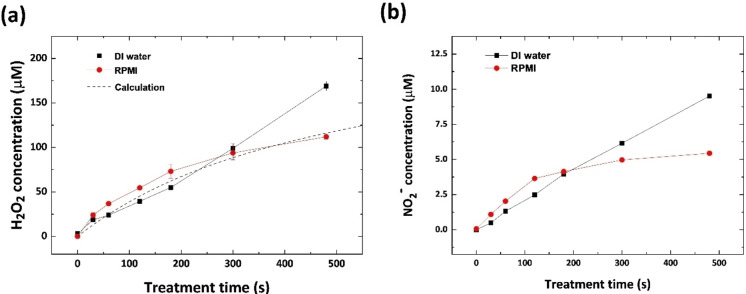
(a) H_2_O_2_ concentrations of the PFLs (black: DI H_2_O, red: RPMI 1640) as functions of treatment time and predicted values based on numerical calculations (dashed line). (b) Temporal NO_2_^–^ concentrations of the PFLs.

Figure 5a and b illustrate the temporal H_2_O_2_ and NO_2_^−^ increases depending on the liquid type. For the two species, the increase rate of the RPMI 1640 PFL is higher than that of the DI water PFL for the first 200 s. Subsequently, the increase rate of the RPMI 1640 PFL noticeably reduces. As discussed previously, the RPMI 1640 PFL has advantages for RONS production, exhibiting high NO and OH intensity, which explains the behavior during the first 200 s. However, H_2_O_2_ scavengers exist in the RPMI 1640 solution and they undergo the following reactions:[^55^, ^56^, ^57^]

(9) $$H_{2}O_{2}+pyruvate\to H_{2}O+CO_{2}+acetate,\;\;k=2.36{\;M}^{-1}\cdot s^{-1}\;$$

(10) $$H_{2}O_{2}+Methionine\to Methionine\;sulfoxide\;k=2\cdot 10^{-2}\;M^{-1}\cdot s^{-1}$$

(11) $$SCH↔{SC}^{-}+H^{+}\;\;\;\;\;\;\;\;\;pKa=8.33\;$$

(11.1) $$H_{2}O_{2}+CS^{-}\to CSOH+OH^{-}\;\;\;\;\;k=15.2{\;M}^{-1}\cdot s^{-1}$$

(12) $$H_{2}O_{2}+NO_{2}^{-}+H^{+}\to ONOO^{-}+H^{+}+H_{2}O\;\;k=8.3\cdot 10^{3}{\;M}^{-2}\cdot s^{-1}\;$$

where SCH is cysteine, and SC^−^ is the negative cysteine ion.

To elucidate the decrease in the rate of H_2_O_2_ generation, numerical chemical calculations for RPMI 1640 were conducted using the ode45 function in MATLAB.^[58]^ This function is a solver for ODEs based on a Runge‐Kutta method, which exhibits a high accuracy with a variable time step, and the calculated values are indicated by the dashed line shown in Figure 5a. The initial conditions of the reactants are as follows: 15 mg/L methionine, 65 mg/L cysteine, and 1 mM sodium pyruvate, and the NO_2_^−^ concentration is set to the experimental data.^[59]^ The rate of H_2_O_2_ generation is 6×10^−7^ M/s, based on the slope of H_2_O_2_ generation. This value is fixed because no changes are observed in the OE spectrum, including the OH peak, and it can be assumed that OH generation in the gaseous phase does not change. According to the comparison between the experimental and calculated values, the decreases in the rate of H_2_O_2_ generation can be caused by scavengers within RPMI 1640, not by the saturation effect.

**Figure 6:**
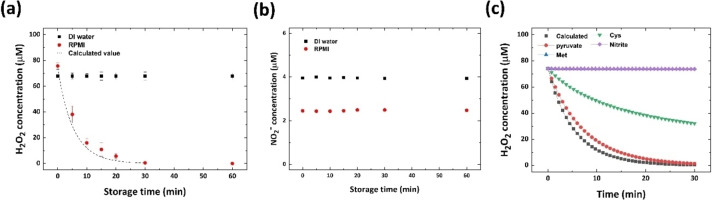
(a) Changes in the concentrations of H_2_O_2_ during the post‐discharge period and the calculated values (dashed line). (b) Changes in the concentrations of NO_2_^–^ during the post‐discharge period. (c) Contributions of candidate scavengers in H_2_O_2_ reduction in the RPMI 1640 solution.

However, as RPMI 1640 contains abundant chemical compounds, including 100 mg/L NO_3_^−^, the plasma‐treated RPMI 1640 solution may be saturated with N. The decrease in the rate of NO_2_^−^ generation may be attributed to the presence of NO_2_^−^ scavenging components within RPMI 1640. However, Figure 6b shows that the NO_2_^−^ scavenging effect is not observed. In contrast, the concentrations of the RONS within the DI‐H_2_O PFL increase steadily with no saturation of acceleration.

### 2.4 Chemical Reactions in PFL Post‐Discharge

Under certain conditions, instead of direct plasma treatment of the media containing cells, the cells are cultured in previously plasma‐treated media.[^60^, ^61^, ^62^] In such a case, as the components and ratio of RONS change with time after plasma treatment, the lapsed‐time limit and optimal lapsed time should be determined. Figures 6a and b show the time‐dependent changes in the RONS within 3‐min‐plasma‐treated PFLs. Although the concentration of NO_2_^–^ remains steady for 60 min, regardless of the liquid type, concentration of H_2_O_2_ is rapidly reduced in RPMI 1640, and the possible causes of H_2_O_2_ reduction are reactions 9–12. For more accurate verification that these reactions are more strongly associated with H_2_O_2_ reduction than the reduction in the concentration of other species, post‐discharge numerical chemical calculations for RPMI 1640 were conducted. The calculated values are indicated by the dashed line shown in Figure 6a. The initial conditions of the reactants are as follows: 15 mg/L methionine, 65 mg/L cysteine, 1 mM sodium pyruvate, and 2.4 μM NO_2_^–^ with 78 μM H_2_O_2_ (the concentrations of which are observed in the 3‐min‐plasma‐treated RPMI 1640). The calculated values are within the margins of error of the experimentally obtained values. Figure 6c shows the corresponding chemical component contributions in H_2_O_2_ scavenging. H_2_O_2_ decreases when only two materials, along with H_2_O_2_, are in the PFL. The duration of 30 min was inadequate for the reaction between H_2_O_2_ and NO_2_^–^ and methionine. Pyruvate, which is recognized as an H_2_O_2_ scavenger, enables the most effective H_2_O_2_ elimination within 20 min, and cysteine is the second major component.

Sodium pyruvate within RPMI 1640 is a special component of the media, and typical RPMI does not contain it. Thus, when H_2_O_2_ is stored in the PFL for >20 min, the use of a sodium‐pyruvate‐free version of RPMI 1640 is recommended.

## 3 Conclusions

The changes in the discharge characteristics and RONS (H_2_O_2_, NO_2_^–^) generation in the PFL due to the liquid type were investigated. Our key findings are as follows:

1. For RPMI 1640, with a high electrical conductivity, the PFL system dissipated more power because the electrode‐like liquid surface produced high concentrations of NO and OH^*^ in the gaseous phase, which could be converted to NO_2_^–^ and H_2_O_2_.
2. H_2_O_2_ generation within RPMI 1640 was strongly limited by scavengers, such as methionine, cysteine, and pyruvate. For numerous biomedical applications wherein the APPJ is utilized, electric shock or RONS generation or stimulation in liquid are considered major steps in realizing goals such as gene transfection, bacteria deactivation, and the stimulation of cell mobility.

Thus, the results of this study may guide the selection of appropriate media, considering the conductivity of the liquid and the influences of scavengers. Furthermore, studying the limitations of the preservation periods of the RONS can be the foundation for determining the response times of APPJ applications. This report provides fundamental data regarding the role of the liquid interface as an electrode and its effect on plasma discharge. Although the focus of this work was a comparison between RPMI 1640 and DI H_2_O, the method can be applied to other cell culture media, plant cultivation solutions, or skin‐like hydrogels. To expand the application scope of the APPJ to various solutions, a fundamental theory for understanding the phenomena is required, and such a theory is presented in this study.

## Conflict of interests

The authors declare no conflict of interest.

4

## Data Availability

The data that support the findings of this study are available from the corresponding author upon reasonable request.
